# Validity and Reliability of a Food Frequency Questionnaire (FFQ) to Assess Dietary Intake of Preschool Children

**DOI:** 10.3390/ijerph16234722

**Published:** 2019-11-27

**Authors:** Yatiman Noor Hafizah, Lee Choo Ang, Fendy Yap, Wan Nurul Najwa, Whye Lian Cheah, Abd Talib Ruzita, Farra Aidah Jumuddin, Denise Koh, Julia Ai Cheng Lee, Cecilia A. Essau, Sue Reeves, Carolyn Summerbell, Edward Leigh Gibson, Bee Koon Poh

**Affiliations:** 1 Nutritional Sciences Programme & Centre for Community Health Studies, Faculty of Health Sciences, Universiti Kebangsaan Malaysia, Kuala Lumpur 50300, Malaysia; hafizahyatiman@ukm.edu.my (Y.N.H.); angleechoo27@gmail.com (L.C.A.); fendy_yap@hotmail.com (F.Y.); najwawannik@gmail.com (W.N.N.); rzt@ukm.edu.my (A.T.R.); farra_aidah@ukm.edu.my (F.A.J.); 2 Department of Community Medicine and Public Health, Faculty of Medicine and Health Sciences, Universiti Malaysia Sarawak (UNIMAS), Kota Samarahan 94300, Sarawak, Malaysia; wlcheah@unimas.my; 3 Sports and Recreation Programme & Centre for Education and Community Well-being, Faculty of Education, Universiti Kebangsaan Malaysia, Bangi 43600, Selangor, Malaysia; denise.koh@ukm.edu.my; 4 Faculty of Cognitive Sciences and Human Development, Universiti Malaysia Sarawak (UNIMAS), Kota Samarahan 94300, Sarawak, Malaysia; aclee@unimas.my; 5 Department of Psychology, University of Roehampton, London SW15 4JD, UK; C.Essau@roehampton.ac.uk (C.A.E.); L.Gibson@roehampton.ac.uk (E.L.G.); 6 Department of Life Sciences, University of Roehampton, London SW15 4JD, UK; S.Reeves@roehampton.ac.uk; 7 Department of Sport and Exercise Sciences, Durham University, Durham DH1 3HN, UK; carolyn.summerbell@durham.ac.uk

**Keywords:** child, dietary assessment, diet records, food frequency questionnaire, Malaysia, methodological study, preschool, reliability, validity

## Abstract

As there are few food frequency questionnaires (FFQ) to assess the dietary intake of preschool children, this study examined the validity and reliability of an FFQ for this purpose. A total of 210 preschoolers aged 4 to 6 years participated in the validation study, while a subsample of 66 participants joined the reliability study. The FFQ is modified from the ToyBox-study and South East Asian Nutrition Surveys (SEANUTS), and comprised 108 food items from 13 food groups. A three-day estimated dietary record (3DR) was used as reference and reliability was assessed through a second administration of the FFQ (FFQ2), four weeks after the first administration (FFQ1). For the validation study, Spearman’s correlation coefficients showed moderate to high correlations (*p* < 0.001) between FFQ and 3DR. Cross-classification of quartile analysis showed moderate agreement between the two methods. As for reliability, Spearman’s correlation coefficients showed moderate to high correlations (*p* < 0.001) between FFQ1 and FFQ2. Cronbach’s alpha values (0.708 to 0.824) and intraclass correlation coefficients (0.710 to 0.826) showed good agreement between repeated FFQs. The results suggest that the FFQ has acceptable validity and good reliability. Hence, the FFQ can be used to assess preschool children’s food intake.

## 1. Introduction

As childhood obesity increases alarmingly worldwide, it is more important than ever to accurately assess the food intake of children, especially from the early to preschool years. In the preschool years, a nutritious diet is critical for children’s growth and development, including cognitive functions [1,2]. To be able to monitor the nutritional status of children, the accurate assessment of food and beverage intakes is crucial [3,4]. An accurate assessment of nutritional status can help identify unhealthy diets and childhood dietary problems that may contribute to both short- and long-term malnutrition [5,6]. In addition to a monitoring function, dietary assessment is an important first step in planning any type of individual or public health dietary intervention. Dietary assessment in preschoolers is particularly challenging because their dietary patterns change more rapidly and more frequently compared to the dietary patterns of other age groups [7].

Early indicators of dietary habits are important because they help in the timely initiation of interventions. Choosing a method for dietary assessment depends on the purpose for which it is intended, and the population group. The food frequency questionnaire (FFQ) is a practical and efficient approach to assess regular diets over time [8]. It is commonly used to assess dietary nutrients intake in epidemiological studies, including total energy intake, selected food or nutrient intakes [9]. FFQs, as for all dietary assessment methods, need to be tailored to the target population’s habitual dietary intake; individual dietary habits are greatly dependent on the ethnic, social, and cultural background of the studied populations [10].

When developing a new dietary assessment tool, in this case a new FFQ, methodological studies which assess the validity and reliability of the tool should be conducted. FFQ validation studies are important because an FFQ with low validity will result in invalid dietary information, leading to inaccurate and unreliable diet and disease associations [11]. According to Mascarenhas et al. [12], FFQs are typically validated by comparing the results with reference methods, such as 24-h diet recalls or dietary records. Dietary records, which are more accurate than other methods, provide information about absolute and relative intakes [9]. In addition, reliability refers to the ability of a method or tool to produce the same results when the measurement is repeated [13]. As dietary measurements with low reliability will produce various errors [14], it is necessary to evaluate the validity and reliability of FFQ for the assessment of nutritional intake in a targeted population.

This study is part of ToyBox Study Malaysia [15], which is a feasibility study to apply healthy lifestyle intervention first designed for European preschool children. The original ToyBox-study used an FFQ developed and validated by Mouratidou et al. [16] to assess the food intake of European preschool children. To adapt the study for use in Malaysia, we modified the European ToyBox Study FFQ by merging it with the South East Asian Nutrition Surveys (SEANUTS) study FFQ, validated by Fatihah et al. [17].

Only two FFQs have been developed specifically for use with Malaysian children [17,18]. Fatihah et al. [17] developed and validated an FFQ for a national survey that included multi-ethnic primary school-aged children, while Nurul-Fadhilah et al. [18] developed one that was targeted towards Malay adolescent populations. Reiterating the importance of a valid and reliable instrument for assessing preschool children’s dietary intakes, this study aimed to validate and assess the reliability of an FFQ that had been specifically adapted and modified for Malaysian preschool children.

## 2. Materials and Methods

### 2.1. Development of the FFQ

The FFQ used in this study was adapted from the original ToyBox-study conducted in Europe by Mouratidou et al. [16] and the FFQ developed and validated by Fatihah et al. [17]. Mouratidou’s FFQ [16] had to be adapted because it was developed for European preschool children and its main purpose was to assess energy intake and the consumption of low energy-density (e.g., fruit and vegetables) and high energy-density (e.g., sugar sweetened drinks, cakes, and biscuits) foods. In contrast, Fatihah’s FFQ [17] was developed for Malaysian children; however, these were school-aged children.

The list of food items from the original ToyBox-study was first reviewed for suitability for use in Malaysian culture prior to merging with the list from Fatihah’s FFQ developed for the SEANUTS Malaysia study. Basically, this study followed the concept of the original ToyBox-study FFQ, which comprised 13 food groups to meet the objectives of the ToyBox-study. The ToyBox-study FFQ food items were merged with those from the SEANUTS FFQ since it consists of food items that are usually consumed by Malaysia children. A preliminary study was then conducted to identify additional commonly consumed foods by Malaysian preschool children, especially by those from the same food culture background. Finally, the FFQ consisted of 108 food and beverage items, which were categorized into 13 food groups, similar to the original ToyBox-study FFQ. The 13 main food groups are: (a) cereals and cereal products; (b) meat and meat products; (c) fish and seafood; (d) eggs; (e) legumes and legume products; (f) milk and dairy products; (g) vegetables; (h) fruits; (i) confectionary; (j) beverages; (k) soup; (l) spreads; and (m) seasonings and flavourings. However, the names of the food groups varied because of cultural differences and food availability. The response for each item consists of eight categories of intake frequency, framed over the past month: never; 1–3 times per month; once a week; 2–4 times per week; 5–6 times per week; once a day; 2–3 times per day; and ≥4 times per day. Household units were used for each food item, such as a scoop, plate, bowl, cup, and tablespoon. The portion size section in the FFQ was designed to reduce the burden on parents or caregivers when completing the questionnaires.

### 2.2. Study Design and Sampling

This methodological study was conducted among preschool children in three different states in Malaysia, namely, Kuala Lumpur, Johor, and Sarawak. These three states were chosen as their population composition is similar to the ToyBox Study Malaysia population target [15]. It is important to note, however, that participants in this study were not the same participants as those who participated in the ToyBox Study Malaysia intervention program, although they share similar cultural backgrounds.

Ethical approval for this study was granted by the Research Ethics Committee of the Universiti Kebangsaan Malaysia (UKM) and the Universiti Malaysia Sarawak (UNIMAS). Permission was also given by the Community Development Department (KEMAS) under the Ministry of Rural and Regional Development, and the Department of National Unity and Integration (Perpaduan) under the Prime Minister’s Department, to recruit in the kindergartens under their management. Kindergartens under KEMAS and Perpaduan in Kuala Lumpur, Johor and Sarawak were randomly selected, and approached for permission to recruit. 

Participants were purposively sampled from a total of 14 randomly selected kindergartens, i.e., four in Kuala Lumpur, five in Segamat, Johor, and five in Kuching, Sarawak. Participants inclusion criteria were Malaysians, aged 4 to 6 years, free from any physical and mental disabilities and with parents or guardians who could read and write, since the FFQ needed to be completed by the primary caregiver. The exclusion criteria were preschool children with special needs and those who were absent from school during data collection or whose parents did not provide consent. The preschoolers involved in this study were from Malay, Chinese, Indian, Iban, and Bidayuh ethnicities.

Information sheets and consent forms were then distributed to the parents to obtain their consent for the child to participate in the study. Questionnaires were completed by their parents or caregivers as proxies.

### 2.3. Anthropometric Measurements

Anthropometric measurements were done according to the protocol from the International Society for the Advancement of Kinanthropometry (ISAK). Height was measured to the nearest 0.1 cm by using Stadiometer SECA Bodymeter 213 (SECA, Hamburg, Germany). Body weight was measured to the nearest 0.1 kg by using SECA Digital Weighing Scale 880 (SECA, Germany). The children were asked to remove as much outerwear as reasonably possible, as well as their shoes and socks and to empty their pockets before their weight was measured. Mid upper arm circumference (MUAC) was measured using Lufkin measuring tape W606PM (Apex Tool Group, Santiago de Querétaro, Mexico) to the nearest 0.1 cm. Body mass index (BMI) was calculated as weight (kg) divided by height squared (m²).

### 2.4. Food Frequency Questionnaire (FFQ)

Parents and caregivers were asked to fill in the foods that the children consumed and the frequency of intake over the past one month. Clear instructions and pictures of common household measures and food portion photographs for each food item were provided. In addition to the 108 food items, the participants had the option to fill in a section named ‘Others’ in the items column, if there were any foods or beverages that did not appear on the FFQ food list. Approximately 2 to 4 weeks after the first administration of FFQ (FFQ1), 66 participants were asked to fill out the FFQ (FFQ2) again to evaluate the FFQ’s reliability.

### 2.5. Three-Day Dietary Record (3DR)

3DR was used as a criterion reference method for the validation of the FFQ. The 3DR was administered for three days (two weekdays and one weekend day). The same parent or caregiver who had completed the FFQ was asked to complete the 3DR. The 3DR was an estimated dietary record and contained sections for the time for each meal’s intake, the food and beverage consumed by their child, and the quantity of the food. Parents or caregivers were asked to provide detailed descriptions of the food and beverages consumed, the food preparation method, and the brand of the food and beverages consumed.

### 2.6. Analysis of Nutrient Intake

The amounts and frequencies of food intakes recorded in the FFQ and 3DR were first converted to grams and then to nutrient intake. Nutrient intakes were estimated using the Malaysian Food Composition Database [19], Atlas of Food Exchanges and Portion Sizes [20], and the Singapore Food Composition database [21]. For food items that were not available in the published literature mentioned above, nutritional food labels and recipes from websites were used as references. The amount of daily food intake was calculated from the FFQ according to the following formula [22]: {frequency of intake (conversion factor) × serving size × total number of servings × weight of food in one serving}. The conversion factor used to estimate food intake was based on the frequency of intake [23]. For dietary data from the FFQ, the total energy and nutrients intake were calculated using an Excel-based platform. The dietary data from the 3DR were analyzed using Nutritionist Pro™ (Axxya System, Woodinville, WA, USA) software to obtain energy and nutrient values for each participant.

### 2.7. Statistical Analysis

All statistical analysis was performed using IBM^®^ SPSS Statistics version 23 (Chicago, IL, USA). Before statistical tests were carried out, the normality of the data in this study was tested using the Shapiro–Wilk test with the 0.05 significance level. Descriptive analysis was used to obtain the frequency, percentage, mean and standard deviation of sociodemographic, anthropometric and dietary intake data. Descriptive statistics of daily energy and nutrients intake calculated from the FFQ and 3DR were presented as mean values ± standard deviation (SD). Wilcoxon signed-rank tests were used to compare values obtained from the FFQ and 3DR and values obtained from FFQ1 and FFQ2, as the data were not normally distributed.

For the validity assessment of the FFQ, energy and macronutrients intake derived from the FFQ were compared with 3DR using Spearman’s correlation coefficient. In addition, the Bland–Altman plot was used to graphically examine the agreement between the FFQ and 3DR for energy and macronutrient intake [24]. The Bland–Altman plots show the differences in intake between the measurement methods by the two methods (FFQ − 3DR; y-axis), against the mean intake of the two measures ((FFQ + 3DR)/2; x-axis). A cross-classification of quartile analysis was used to examine agreement between the two tools in terms of proportion of participants’ energy and nutrient intakes. Cross-classification analysis was done by classification of participants into quartile categories based on the dietary intake data from both the FFQ and 3DR.

For the reliability of the FFQ, Spearman’s correlation was used to assess the relationship between FFQ1 and FFQ2. Cronbach’s alpha values were calculated to assess the agreement and reliability between FFQ1 and FFQ2. In addition, intraclass correlation coefficients (ICCs) were calculated, comparing the first (FFQ1) and second administration (FFQ2) to assess the reliability [25].

## 3. Results

Of the 241 participants who agreed to participate in the study, only 210 preschool children (103 boys, 107 girls) completed the study protocol. A total of 25 participants were excluded as they were unable to complete both the FFQ and 3DR and 6 participants were excluded after examination of outliers. Table 1 shows the physical characteristics of participants. Mean age was 5.2 ± 0.7 years old. Boys had similar anthropometric characteristics and were not significantly different from girls.

Energy and macronutrient intake data obtained by the FFQ were significantly higher than those reported by 3DR (Wilcoxon signed-rank tests, all *p* < 0.001) (Table 2). Spearman’s correlations showed moderate to high positive correlations between the two methods, with correlations ranging from r_s_ = 0.363 (fat) to r_s_ = 0.511 (energy), *p* < 0.001.

The mean intake of energy and macronutrients and the mean difference and correlation between FFQ1 and FFQ2 are shown in Table 3. The mean intakes of energy and macronutrients in FFQ1 were not significantly different from those in FFQ2 (Wilcoxon signed-rank tests). Positive Spearman’s correlation coefficients ranged from r_s_ = 0.464 (carbohydrate) to r_s_ = 0.665 (protein) (all *p* < 0.001). These findings indicate moderate to high consistency between FFQ1 and FFQ2.

The reliability of the FFQ was also assessed using the Cronbach’s alpha and ICC (Table 3). Cronbach’s alpha values for the energy and macronutrients intake based on FFQ1 and FFQ2 indicated good agreement that ranged from 0.708 for carbohydrate to 0.824 for fat. ICC showed moderate to high reliability between the repeated FFQs. The ICC coefficients for the intake of energy and macronutrients ranged from 0.710 (protein) to 0.826 (fat).

Table 4 shows that the percentages of participants classified into the same and adjacent quartiles ranged from 77.6% (fat) to 83.3% (carbohydrate), with gross misclassification occurring in 16.7% to 22.4% of participants.

**Figure 1:**
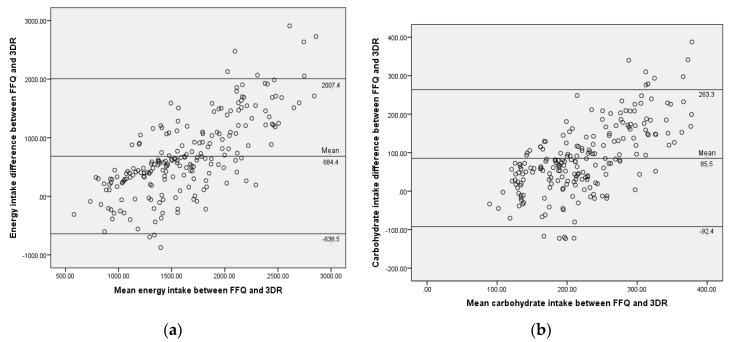

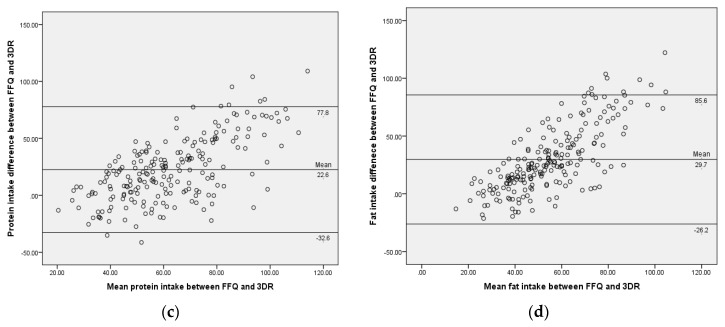
Bland-Altman plots show agreement between the FFQ and 3DR for (**a**) energy; (**b**) carbohydrate; (**c**) protein; and (**d**) fat intake. The solid line represents the mean difference between the FFQ and 3DR, and the dashed lines represent the limits of agreement corresponding to ±1.96 (SD).

The Bland–Atman method showed a positive mean difference for energy and macronutrients, indicating that there is overestimation of energy and nutrient intakes by the FFQ as compared to the 3DR (Figure 1).

## 4. Discussion

The FFQ has not been used extensively in studies of preschool children but it is gaining importance due to the increasing prevalence of overweight and obesity among children. This study describes the validation of an FFQ specifically designed to assess the diet of Malaysian preschool children, when completed by their primary caregiver. The FFQ was adapted from the European ToyBox-study and SEANUTS [16,17]. Both FFQs were developed to target the age group of 4 to 6 years old. While meeting the objectives of ToyBox-study, the FFQ was merged with SEANUTS FFQ to meet the Malaysian cultural food choices. Thus, the validity of the modified FFQ was tested by comparing nutrient intakes with those assessed by a 3-day diet record (3DR), while reliability was measured by repeating the administration of the FFQ with a subsample of participants. In terms of validity, the mean nutrient amounts from the FFQ were higher than those obtained with the 3DR.

Assessment of mean differences reflects the agreement between the two methods. Based on Lombard et al. [26], the percentage difference of 0 to 10.0% indicates an acceptable outcome, while a percentage difference of more than 10% indicates a poor outcome. Results showed that the percentage difference for the energy and macronutrient intakes were more than 10%. The result of this study is consistent with Fatihah’s study [17] of Malaysian children aged 7 to 12 years. The study showed mean differences of more than 10% for the intakes of energy and macronutrients ranging from 11.9% (fat) to 30.8% (carbohydrate). Furthermore, a study of Polish children aged 3 years old, found differences of 16.5% for carbohydrate and 14% for fat [27]. This is also in line with the literature on dietary assessments which typically find that FFQs tend to overestimate dietary intakes, whereas diet diaries tend to underestimate food intake [28].

Cross-classification and Bland–Altman plots were used to assess the relative validity of the FFQ against the 3DR. The two methods showed moderate agreement between the FFQ and the 3DR. Cross-classification analyses, where energy and nutrient intakes were classified into the same or adjacent quartiles or misclassified in the opposite quartiles, were conducted to validate the agreement between the two methods [29]. In this study, over 10% of the participants were grossly misclassified, based on their energy and macronutrient intakes. According to Masson et al. [25], when more than 10% of participants were grossly misclassified in the opposite quartiles, it demonstrates a poor outcome. On the other hand, more than 70% of the participants in the study were classified into the same or adjacent quartiles, suggesting that the modified FFQ had achieved a satisfactory level of agreement. Fatihah et al. [17] arrived at similar results in a previous study, where 70.3% (fat) to 84.2% (energy) of their participants were correctly classified into the same or adjacent quartiles. 

The Bland–Altman plot was used to assess the agreement between the FFQ and the 3DR. The tendency towards overestimating the data from FFQ is reflected graphically in the Bland–Altman plot. This tendency of the FFQ to overestimate dietary intake was also reported in a previous study by Fumagalli et al. [30], indicating that the FFQ appears to overestimate food intakes of children aged 5 to 10 years. The closer the mean of the differences is to zero and the narrower the agreement interval is, the better the agreement between the two tools [24]. The Bland–Altman plots showed that there is less satisfactory agreement between the FFQ and the 3DR due to the wide agreement interval and overestimation of the FFQ. However, this could be because although the 3DR is often used as the reference method, it is not the “gold standard”; much of the difference between the FFQ and 3DR may be due to underreporting by the 3DR [31].

Overall, moderate correlations were found between the FFQ and the 3DR. Spearman’s correlations of 0.30–0.49 are considered ‘acceptable’ and considered ‘good’ if they are over 0.50 [32]. The correlation coefficients between the FFQ and the 3DR were considered moderate to good. The finding is similar to results from a validity and reliability FFQ study of Japanese children aged 6 years [33]. The validity correlation ranged from r_s_ = 0.34 (energy) to r_s_ = 0.40 (protein). Weaker correlation for total fat intake was also reported in the study by Fumagalli et al. [30] among Brazilian children aged 5 to 10 years (r_s_ = 0.153).

For the reliability of the FFQ, nutrient estimates were compared between the first (FFQ1) and second FFQ (FFQ2) administrations. According to McPherson et al. [34], in reliability studies, food intake assessed by FFQ often appears to be higher during first administration as compared to the second; this is apparent for example in FFQ reliability studies by Fatihah et al. [17], Huybrechts et al. [13], and Buch-Andersen et al. [35]. However, in our study, nutrient intakes did not differ significantly between first and second administrations, even though energy and carbohydrate intakes were slightly higher for the first FFQ. Assessment of reliability is particularly important for FFQs designed to assess the diet of preschool children, because of the use of parents or guardians as proxies in the assessment of their children’s dietary intake. The estimation of the type and the amount of food consumed by the children can be challenging. Nevertheless, Spearman’s correlations of 0.30 to 0.49 is considered ‘acceptable’ and correlations of 0.50 to 0.70 are considered ‘good’ [13]. The correlations of repeated FFQs were generally similar to the previous studies. For example, in a study conducted by Moghames et al. [6] on Lebanese children aged 5 to 10 years, the correlations ranging from r_s_ = 0.64 (carbohydrate) to r_s_ = 0.77 (energy) were found. Fatihah et al. [17] also reported moderate correlation r_s_ = 0.467 for carbohydrate.

In this reliability study, of a relatively large study group sample, the results demonstrated good agreement between FFQ1 and FFQ2. In order to evaluate the reliability of FFQ, Cronbach’s alpha and ICC were used to assess the agreement between the repeated FFQs. Cronbach’s alpha assesses the variability between the individual nutrient intakes relative to the total variability of intakes. A high coefficient alpha means the tool used is reliable [36]. Cronbach’s alpha value demonstrated good agreement between FFQ1 and FFQ2, as the value is between 0.70 and 0.80 [37]. A previous study by Fatihah et al. [17] also illustrated good reliability between FFQ1 and FFQ2 in which the Cronbach’s alpha ranged from 0.606 (protein) to 0.703 (energy). ICC showed moderate to high reliability between the repeated FFQs. Based on the 95% confident interval of the ICC estimate, value between 0.5 and 0.75 indicates moderate reliability, while value between 0.75 and 0.9 is indicative of good reliability [38].

### Strengths and Limitations

The limitations of using a parent-proxy report FFQ to assess the dietary intake of young children are acknowledged. The food intake frequency reporting may be limited to only when the children are at home or with their parents or main caregivers who are proxy-reporting their intake. It is difficult to estimate intake for the children when they are in childcare or when they are being cared for by a babysitter. Providing separate questionnaires to other caregivers, such as childcare staff, may help to improve accuracy and could be considered for future studies. 

This study collected dietary data in the form of two FFQs and a 3DR; both methods have their own limitations not least because they rely upon self-report data [8]. We acknowledge that the 3DR is not a gold standard although it is often used as the reference method [11]. The literature on validation studies recommends additional methods such as the motion sensor measurement of total energy expenditure as an independent measure to reflect total energy intake. The 3DR method does not have correlated errors with the test method (FFQ). Whilst biomarkers are considered the superior reference method (e.g., 24-h urinary collection of recovery biomarkers or blood samples for concentrations markers), the collection of such data is difficult in the preschool age group studied here. Furthermore, the relationship between dietary intakes of a nutrient and its biomarker are not always direct and may be influenced by absorption [39].

One of the major strengths of this study is the large study-sample, which is actually larger than in most comparable validation studies in other countries. Another strength is the demographic characteristics of the study sample, which included ethnic, cultural, and socio-economic diversity that reflect the composition of the Malaysian population.

## 5. Conclusions

The findings from this study suggest that the FFQ has good reliability and acceptable validity when compared to a 3-day dietary record. The FFQ will be useful in the assessment of food intake by preschool children in Malaysia, such as in the ToyBox Study Malaysia. It is recommended that interview-administration when applying the FFQ in future studies will help to improve the validity of the FFQ. Further evaluation and modifications of food items may be needed to tailor the food items in the FFQ for preschool children in other states of Malaysia as their food culture may be different from those living in Kuala Lumpur, Johor, and Sarawak.

## Figures and Tables

**Table 1:** Physical characteristics of participants (*n* = 210).

Characteristics	Total (*n* = 210)	Boys (*n* = 103)	Girls (*n* = 107)	*p* Value
	Mean ± SD			
Age (years)	5.2 ± 0.7	5.2 ± 0.7	5.2 ± 0.7	0.615 ^a^
Weight (kg)	18.1 ± 4.1	18.6 ± 4.6	17.7 ± 3.5	0.408 ^a^
Height (cm)	107.1 ± 7.0	107.3 ± 7.3	106.9 ± 6.6	0.392 ^b^
Body mass index (BMI) (kg/m^2^)	15.7 ± 2.7	16.1 ± 3.1	15.4 ± 2.2	0.269 ^a^
Mid upper arm circumference (MUAC) (cm)	16.7 ± 2.5	17.1 ± 2.7	16.4 ± 2.2	0.136 ^a^

^a^ Mann–Whitney test was used to examine difference between the sexes. ^b^ Independent t-test was used to examine difference between the sexes.

**Table 2:** Mean ± SD, mean difference and Spearman’s correlation (r_s_) for energy and macronutrients intake obtained using Food frequency questionnaire (FFQ) and Three-day dietary record (3DR) (*n* = 210).

Nutrients	FFQ	3DR	Mean Difference	% of Mean Difference	Spearman’s Correlation (r_s_)
	Mean ± SD	Mean ± SD			
Energy (kcal)	1978 ± 769	1293 ± 333	685 ^a^	53.0	0.511 **
Carbohydrate (g)	265.8 ± 102.5	180.3 ± 50.2	85.5 ^a^	47.4	0.498 **
Protein (g)	74.0 ± 30.7	51.4 ± 14.9	22.6 ^a^	44.0	0.439 **
Fat (g)	69.8 ± 30.3	40.1 ± 11.7	29.7 ^a^	74.1	0.363 **

Percentage mean difference was individually calculated using the formula (mean FFQ − mean 3DR)/mean FFQ × 100% [23]. ^a^ Wilcoxon signed-rank test showed significant differences between FFQ and 3DR, *p* < 0.001. ** Spearman’s correlations were all significant at *p* < 0.001.

**Table 3:** Mean ± SD, mean difference, Spearman’s correlation (rs), Cronbach’s alpha values (α), and intraclass correlation (ICC) for energy and macronutrients intake obtained using FFQ1 and FFQ2 in the subsample (*n* = 66).

Nutrients	FFQ1	FFQ2	Mean Difference	% of Mean Difference	Spearman’s Correlation (rs)	FFQ1 vs. FFQ2	
	Mean ± SD	Mean ± SD				α	ICC
Energy (kcal)	2084 ± 806	2048 ± 755	−36	−1.7	0.630 **	0.823	0.825 **
Carbohydrate (g)	284.6 ± 105.4	277.3 ± 99.7	−7.3	−2.6	0.464 **	0.708	0.710 **
Protein (g)	78.3 ± 35.5	79.0 ± 33.7	0.7	0.9	0.665 **	0.791	0.794 **
Fat (g)	72.5 ± 31.8	72.3 ± 29.9	−0.2	−0.3	0.655 **	0.824	0.826 **

Percentage mean difference was individually calculated using the formula (mean FFQ2 − mean FFQ1)/mean FFQ1 × 100% [6]. Wilcoxon signed-rank tests found no significant differences between FFQ1 and FFQ2. ** Spearman’s correlations, *p* < 0.001.

**Table 4:** Cross-classification for energy and macronutrients based on FFQ and 3DR (*n* = 210).

Nutrients	Cross-Classification between FFQ and 3DR		
	% CC	% CCI	%GM
Energy (kcal)	46.7	79.0	21.0
Carbohydrate (g)	42.4	83.3	16.7
Protein (g)	33.3	79.5	20.5
Fat (g)	37.1	77.6	22.4

% CC = percentage of participants with FFQ and 3DR intakes that correctly classified in the same quartiles. % CCI = percentage of participants with FFQ and 3DR intakes that classified in the same and adjacent quartiles. % GM = percentage of participants with FFQ and 3DR intakes that grossly misclassified in the extreme quartiles.

## Abbreviations

The following abbreviations are used in this manuscript:

FFQ	Food frequency questionnaire
3DR	Three-day dietary record
ICC	Intraclass correlation coefficients
CC	Correctly Classified
CCI	Correctly Classified Instance
GM	Grossly Misclassified
